# Galvanism Applied to the Treatment of Uterine Hæmorrhage, &c.

**Published:** 1845-05

**Authors:** 


*Galvanism applied to the Treatment of Uterine Hæmorrhage, &c.*—Dr. Radford says that he has pursued this practice with great success, in cases of hæmorrhage, accidental or unavoidable, accompanied by exhaustion, and occurring before, during, or after labor. He adds—

"I am satisfied, from positive trial of the remedy, that it will be found a most important agent in tedious labor, depending upon want of power in the uterus, and where no mechanical obstacle exists. I would also suggest the probability of its proving valuable in originating uterine action *de novo*, in cases where it may be considered necessary to induce premature labor. It seems to me also to be worthy of trial in certain cases of menorrhagia in the ungravid state, where, on vaginal examination, the uterus is found to be atonic, as evidenced by its large flaccid condition, and the patulous state of the os uteri."

The remedy is thus applied:—

"The brass ball of the vaginal conductor is to be passed up to the os uteri, and moved about, at intervals, on to various parts of this organ; and, at the same time, the other conductor must be applied to the abdominal parietes over the fundus uteri. Shocks may be also passed transversely through the uterus, by simultaneously applying the conductor on each side of the belly.

"The application should be used at intervals, so as to approximate in its effects as nearly as possible, to the natural pains. It may be continued until it meets the exigencies of the case."—*Lancet*, in *Bulletin of Med. Science.*

